# Associations between social anxiety and avoidance, attachment styles and parental marital status, in late adolescence

**DOI:** 10.1192/j.eurpsy.2021.484

**Published:** 2021-08-13

**Authors:** B. Rodrigues Maia, C. Coelho, M. Marques, F. Carvalho

**Affiliations:** 1 Faculty Of Philosophy And Social Sciences, Centre For Philosophical And Humanistic Studies, Portugal, Universidade Católica Portuguesa, Braga, Portugal; 2 Faculty Of Philosophy And Social Sciences, Universidade Católica Portuguesa, Braga, Portugal; 3 Coimbra Hospital And University Centre, Portugal, Coimbra Hospital and University Centre, Coimbra, Portugal; 4 Espaço Psicológico – Consultório De Psicologia, Coimbra, Portugal, Espaço Psicológico – Consultório de Psicologia, Coimbra, Portugal, Coimbra, Portugal

**Keywords:** social anxiety and avoidance, attachment styles, parental marital status, late adolescence

## Abstract

**Introduction:**

The relation between insecure general attachment and social anxiety has long been established.

**Objectives:**

To explore the associations between social interaction and performance anxiety and avoidance, attachment styles, and parental marital status.

**Methods:**

146 Portuguese adolescents, with a mean age of 18.99 years old (SD = .848; range: 18-20), filled in the Social Interaction and Performance Anxiety and Avoidance Scale and the Experiences in Close Relationships-Relationship Structures Questionnaire.

**Results:**

Distress/Anxiety was correlated with avoidance attachment to mother and father (rs = .17*, p = .04; rs = .18*, p = .03), to anxious attachment to romantic partner (rs = .21*, p = .01), and to anxious and avoidance attachment to best friend (rs = .25**, p = .00; (rs = .17*, p = .035). Avoidance was significantly correlated with avoidance to father and to romantic partner (rs = .18*, p = .03), and to anxious and avoidance attachment to best friend (rs = .21**, p = .009; rs = .18*, p = .03). A significant difference was found in avoidance attachment to father X^2^ = 10.246 (4, n = 146), p = .036, by parental marital status, with the adolescents with single/divorced parents presenting a higher mean score (Md = 111.10; Md = 82.93) than the other groups.

**Conclusions:**

Distress/anxiety seems to be associated with more close relationships, and a single/divorced status with Avoidance. Longitudinal studies are needed to explore if insecure attachment to parents predicts insecure extra-familiar attachment, and to explore the long-term effects of parental marital status.

